# Perturbation recovery during walking is impacted by knowledge of perturbation timing in below-knee prosthesis users and non-impaired participants

**DOI:** 10.1371/journal.pone.0235686

**Published:** 2020-07-13

**Authors:** Matthew J. Major, Chelsi K. Serba, Keith E. Gordon

**Affiliations:** 1 Department of Physical Medicine and Rehabilitation, Northwestern University, Chicago, Illinois, United States of America; 2 Department of Biomedical Engineering, Northwestern University, Evanston, Illinois, United States of America; 3 Jesse Brown VA Medical Center, Chicago, Illinois, United States of America; 4 Edward Hines Jr. VA Medical Center, Hines, Illinois, United States of America; 5 Department of Physical Therapy and Human Movement Sciences, Northwestern University, Chicago, Illinois, United States of America; University of Colorado Boulder, UNITED STATES

## Abstract

Previous research found that below-knee prosthesis users proactively increase their lateral margin-of-stability on their impaired side in anticipation of an impending perturbation when the timing is predictable and potentially directed toward the impaired limb. While knowledge of perturbation timing and direction influences proactive strategies, the consequences of such knowledge and anticipatory behavior on recovery from perturbations is unclear. This study characterized center-of-mass (CoM) dynamics of below-knee prosthesis users and non-impaired controls following a lateral perturbation when the perturbation direction is known but *a priori* knowledge of the timing of perturbation is either known or unknown. Across groups, CoM displacement during perturbation exposure increased when directed towards the impaired or non-dominant limb with no influence of timing knowledge. In addition, peak CoM displacement was less with known timing irrespective of the perturbation direction. Generally, the CoM displacement during perturbation exposure correlated well with the CoM medial-lateral velocity during unperturbed walking, supporting evidence that human response dynamics to lateral perturbations are influenced by the instantaneous state of the body’s momentum.

## Introduction

Maintaining frontal-plane locomotor stability is a known challenge for below-knee prosthesis users (BKPUs) [1, 2]. Control of frontal-plane locomotor stability is suggested to require greater active involvement of the nervous system than sagittal-plane stability [3–5]. For BKPUs the consequences of lower limb loss, including a reduced ability to sense body dynamics [6, 7] and to generate active joint control [8–11], contribute to the challenge of controlling frontal-plane locomotor stability. Considering that approximately 50% of community-living individuals with lower limb loss will fall at least once per year [12] and that the majority of these falls are initiated by intrinsic factors (i.e., those attributable to the person) [13] it is important that we understand the issues influencing the capacity of BKPUs to maintain frontal-plane locomotor stability.

BKPUs and non-impaired individuals use a combination of control mechanisms during walking to maintain the periodic frontal-plane oscillations of their center of mass (CoM) within their base of support [11, 14–16]. In BKPUs (who do not use a powered prosthesis) two important frontal-plane stability control mechanisms, ankle push-off [17, 18] and lateral ankle [17, 19] strategies, will be unavailable because they rely on muscles crossing the ankle joint. In the absence of these active ankle strategies, BKPUs often exhibit gait patterns (e.g. slower walking speeds, and faster, wider steps than their non-impaired counterparts [20–22]) believed to increase frontal-plane locomotor stability [11, 16, 23]. These gait characteristics are modifiable and typically become more pronounced in environments that continuously challenge medial-lateral balance [2, 21, 24, 25]. Dependent on *a priori* knowledge of an impending discrete lateral perturbation [26], BKPUs will also make specific anticipatory gait adaptations. These adaptations include increasing the lateral margin-of-stability (MoS) on the impaired side during the step prior to the perturbation onset if the perturbation was known to be certainly or possibly directed towards the impaired limb [26]. This increase in lateral MoS occurred without significant changes in step width when compared to baseline walking suggesting that to prepare for the perturbation individuals increased their lateral MoS by controlling CoM dynamics rather than modifying their lateral base of support. Similar anticipatory perturbation-specific adaptations were not observed in non-impaired individuals, or when perturbation timing was not known.

It is difficult to evaluate how the cumulative effect of these control mechanisms impact BKPUs’ capacity to respond to discrete lateral perturbations. Thus, the primary aim of this study was to characterize the resulting CoM dynamics of BKPUs wearing passive ankle-foot prostheses and non-impaired controls following discrete lateral perturbations during walking when the direction of the perturbations were known *a priori* but timing knowledge is varied. In light of our previous research finding that knowledge of perturbation timing and direction influenced BKPUs anticipatory strategies [26], we hypothesized that due to lost sensory function and active joint control [6–10], when the perturbation timing is either *known* or *unknown* and directed toward their impaired limb that prosthesis users would exhibit a greater lateral CoM displacement when compared to the effects of perturbations directed towards their sound limb. We also hypothesized that BKPUs’ strategy of increasing their lateral MoS when perturbation timing is both *known and possibly directed* toward the impaired limb [26] would result in smaller CoM displacements during known time perturbations when compared to unknown time perturbations. Furthermore, as the ability to resist a lateral perturbation may reasonably be influenced by gait phase-dependent factors including instantaneous lateral CoM velocity [27] and limb support phase [28], a secondary aim was to characterize CoM displacement as a function of the gait cycle. In this context, inclusion of non-impaired control data provides a reference for which to compare BKPU responses. Results from this work will connect anticipatory strategies with response dynamics. The insight gained into the role of anticipatory locomotor strategies on stability will improve our understanding of the control factors related to increased fall prevalence in lower-limb prosthesis users when sensorimotor function is unilaterally impaired.

## Methods

### Participants

The study protocol was approved by the Northwestern University Institutional Review Board (IRB # STU00200872) and all participants provided informed written consent prior to study involvement. The individual pictured in this manuscript has given written informed consent (as outlined in PLOS consent form) to publish these case details. We recruited both BKPUs and non-impaired participants. Inclusion criteria for all participants included: 18 to 65 years of age, normal/corrected vision, and able to walk continuously for 10 minutes without undue fatigue or health risks. BKPUs were also required to possess a unilateral below-knee amputation, be daily users of their clinically prescribed prosthesis for ambulation, walk in the community without a mobility aid, prosthesis usage for at least one year, and a residual limb in good condition (no scars, ulcers, infections, etc.). Exclusion criteria for all participants included: self-identified musculoskeletal (apart from amputation) and/or vestibular pathologies impairing balance and/or stability, taking medications affecting proprioception and/or balance, and cognitive deficits that preclude understanding of testing instructions.

### Experimental setup

**Fig 1:**
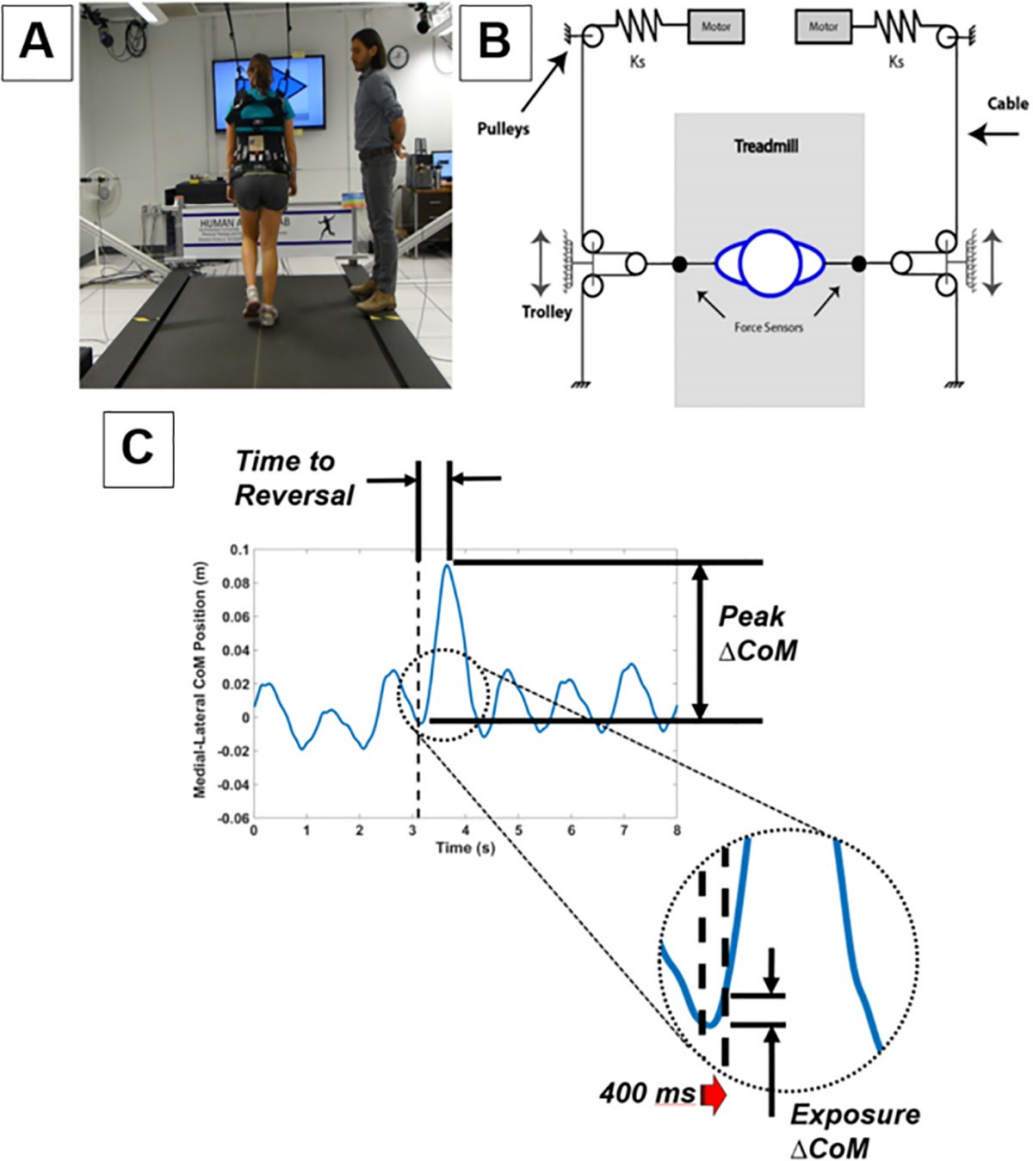
Cable-robot setup (a) and design (b) for applying lateral perturbations to the CoM (adopted from Major et al 2018 [26]), and measured variable definitions (c) according to temporal medial-lateral COM position.

Participants walked on a treadmill (Tuff Tread, Willis, TX) with an oversized belt (1.39m width) that provided space for responding to lateral perturbations. For safety, participants wore a passive overhead harness (Aretech, Ashburn, VA) adjusted so it did not restrict lateral movement. A cable-driven robotic device [29, 30] delivered discrete lateral perturbations during select walking trials (Fig 1A). The robot’s independent series-elastic linear motors modulated tensile loads applied to the medial attachments of a pelvis harness via steel cables (Fig 1B). During all treadmill walking, with the exception of the periods when perturbations were applied, the robotic device operated in a transparent mode—zero net lateral force applied to participants through equal and opposite tensile forces. Perturbation exposures lasted 400 msec and constituted a net unilateral force of 12% bodyweight. The robotic device returned to transparent mode operation following delivery of each discrete perturbation. The perturbation magnitude was selected based on pilot testing to deliver a challenging but recoverable disturbance for BKPUs. Auditory and visual feedback on the timing and direction of each impending perturbation were provided through audio speakers and a 60-inch monitor mounted 1.8 m in front of the treadmill (Fig 1A).

A ten-camera motion capture system (Qualisys, Göteborg, Sweden) recorded 3-D positions (100 Hz) of retro-reflective markers attached to the pelvis (superior iliac crests, anterior-superior iliac spines, spine sacral level 2, and two tracking markers) and bilaterally on the greater trochanter, lateral knee, lateral malleolus, calcaneus, and second and fifth metatarsals. Prosthetic foot marker positions approximately matched the sound foot. The motion capture system also collected synchronized kinetic data from the robotic device’s load cells at 1000 Hz.

### Experimental protocol

Participant demographics and additional health history from BKPUs on prosthesis use (type and experience), time since amputation, amputation etiology, functional balance, and socket comfort were collected first. The Berg Balance Scale, a valid and reliable outcome measure for lower limb prosthesis users [31], and the commonly-used Socket Comfort Score [32] were administered to measure functional balance and comfort of the prosthetic socket, respectively. A staircase method of increasing and decreasing the belt speed while participants walked on the treadmill was then used to identify a preferred walking speed as confirmed through verbal feedback. Walking practice of, on average, two minutes allowed participants to familiarize with the setup and protocol.

Participants performed a series of walking trials at their preferred speed that were grouped into blocks based on participants' *a priori* knowledge of the lateral perturbation:

1. **Baseline**–no perturbations were applied
2. Unknown Time and Unknown Direction
3. **Unknown Time and Known Direction**
4. **Known Time and Known Direction**
5. Known Time and Unknown Direction

* Only **Bold** conditions were analyzed for this experiment. We have previously analyzed this dataset and reported on anticipatory adaptations for all the above conditions [26].

Each block contained six trials. The order of the blocks were randomly presented to minimize order bias. All blocks, except for baseline, were repeated twice to collect equal numbers of right- and left-directed perturbations. Each trial included 20 seconds of continuous walking. Participants received a single discrete lateral perturbation during each non-Baseline trial. Results analyzing the anticipatory locomotor strategies for all five conditions have been published separately [26]. Here we present an analysis of the reactive locomotor strategies from the Baseline (condition 1), Unknown Time and Known Direction (condition 3), and Known Time and Known Direction (condition 4) to address the two main hypotheses and secondary aim concerning the effect of perturbation timing uncertainty when perturbation direction was known. As relevant to this analysis, all perturbations in a block for conditions 3 and 4 (known direction), were in the same direction. Prior to beginning each test block, participants experienced the audio-visual feedback and lateral perturbation relevant to each condition during quiet standing to become familiarized with the protocol.

A large arrow pointing to the right or left was displayed on the monitor to provide knowledge of the perturbation direction (Fig 1A). A visual progress bar timer in the form of a large rectangle that filled from left to right was displayed on the monitor and synchronized with an audible 5-second countdown to provide knowledge of perturbation timing. When timing knowledge was withheld, the progress bar timer would not fill and the countdown was absent. During unperturbed baseline trials, the monitor displayed a question mark and audio timing cues were provided in an effort provide similar audiovisual stimulation across all conditions.

Participants were instructed to walk along the center of the treadmill, defined by a distinct yellow chalk line, and to return to the treadmill center as quickly as possible after each perturbation. Participants were specifically instructed not to consider the chalk line as a “tight-rope.” To minimize fatigue bias participants were allowed to rest between individual trials and test blocks for as long as requested. Although participants regularly rested between blocks, none chose to rest between trials and so each block was tested during continuous walking. However, subsequent trials were started only after the participant was observed to have fully regained steady-state walking (approximately 20 steps). The treadmill was gradually stopped after each block.

### Data analysis

Using Visual 3D software (C-Motion, Germantown, MD), marker position trajectories were low-pass filtered (Butterworth, 6 Hz cut-off frequency), and the CoM position was estimated as the center of a pelvis model built from the pelvis markers. Evidence suggests that this simple approximation of CoM position is comparable to more complex multi-segment models when estimating instantaneous medial-lateral CoM position [33].

Visual 3D was also used to estimate initial foot contact and toe-off events for each limb as the maximum anterior distance between the calcaneus and pelvis center, and maximum posterior distance between the fifth metatarsal and pelvis center, respectively. Accuracy of these event times were manually (visually) confirmed and adjusted as appropriate.

Response dynamics to each perturbation were characterized through three features of the temporal medial-lateral CoM position across a given trial (Fig 1C):

1. ***ΔCoM***_***exposure***_
  CoM displacement in the same direction as the perturbation during the perturbation exposure period of 400 msec (*ΔCoM*_*exposure*_ *= CoM position at perturbation end–CoM position at perturbation onset*);
2. ***ΔCoM***_***peak***_
  Peak CoM displacement in the same direction as the perturbation following the perturbation onset
  (Δ*CoM*_*peak*_ *= Maximum CoM position following the perturbation onset–CoM position at perturbation onset*);
3. **T**_***reversal***_
  Time to reversal of the CoM
  (T_*reversal*_ *= time at maximum CoM position–time at perturbation onset*).

We choose to examine CoM displacement using two methods. *ΔCoM*_*exposure*_ provided information about the displacement during the 400 msec period when the perturbation was applied. This measurement of the displacement provided insight into the immediate resistance of the body to lateral perturbations. In contrast, *ΔCoM*_*peak*_ and T_reversal_ provided information about the control of the CoM following the completion of the perturbation. As such, measures of *ΔCoM*_*peak*_ and T_reversal_ sometimes occurred during the steps following the perturbation step.

Custom MATLAB (Mathworks, Natick, MA) software was used to calculate each of these parameters for a given trial and the average values were calculated for each test condition across the six trials. The two CoM displacement metrics were normalized to body height and replaced with a value of zero in the rare occasion (5.8% of *ΔCoM*_*exposure*_ measurements, and 0.5% *ΔCoM*_*peak*_ measurements of the >445 total trials) that the displacement was slightly in the direction opposite to the perturbation. As relevant to the primary set of hypotheses, the CoM displacements and time to reversal were used to characterize the spatial and temporal response components, respectively. While the temporal component did not directly address our hypotheses, it was considered an important metric to help interpret reactive control.

To address the secondary aim, identifying the influence of gait phase on acute response dynamics, *ΔCoM*_*exposure*_ for each trial across all participants were plotted against the perturbation onset time with respect to the gait cycle starting with the initial contact of the impaired (BKPU) or non-dominant (control) limb. Plots were created with data separated by perturbation direction, group, and timing knowledge, and zero values corresponding to no displacement in the perturbation direction were removed.

### Statistical analysis

To address the primary aim of assessing the direction and timing knowledge effects on CoM response dynamics, a three-way mixed ANOVA (one between-group factor, two within-groups factors) was used to test the main and interaction effects of timing knowledge (known, unknown), direction (towards impaired/sound, non-dominant/dominant), and group (BKPU, control) on *ΔCoM*_*exposure*_, *ΔCoM*_*peak*_, *and T*_*reversal*_. Specifically, analyses of *ΔCoM*_*exposure*_ and *ΔCoM*_*peak*_ addressed the first and second hypotheses, respectively. The absence of violations of normality that would affect the results given robustness of mixed ANOVA was confirmed using the Shapiro-Wilk test and observation of Q-Q plots of the model residuals. Assumptions of homogeneity of variance and covariance were confirmed with the Levene’s test and Box’s test, respectively. Effect sizes were estimated using the partial η^2^ values.

To address the secondary aim of assessing how perturbation response was affected by gait-phase dependent factors that theoretically influence CoM dynamics, we focused on evaluating the relationship between instantaneous lateral CoM velocity and CoM displacement during the exposure period (*ΔCoM*_*exposure*_*)*. Graphical observation of *ΔCoM*_*exposure*_ versus perturbation onset time suggested that for both groups the CoM displacement profile: 1) resembled that of the baseline (unperturbed condition) temporal CoM medial-lateral velocity across the gait cycle, and 2) was not considerably influenced by timing knowledge. Consequently, the data were reduced to four sets separated by group and perturbation direction, and for each dataset a repeated-measures correlation analysis was conducted between *ΔCoM*_*exposure*_ and instantaneous baseline CoM medial-lateral velocity to determine the strength and significance of their relationship across participants while accounting for paired data within participants [34]. As each perturbation occurred at some instance during the gait cycle, this analysis assessed how strongly CoM response dynamics were linked to instantaneous COM velocity at that corresponding time during baseline walking. Prior to this analysis, to account for the 400 msec exposure, each *ΔCoM*_*exposure*_ *data point* was temporally advanced by the percentage of the average baseline stride time corresponding to 200 msec (16% for BKPUs and 19% for controls) representing the temporal midpoint of the perturbation.

The ANOVA and correlation statistical analyses were conducted with SPSS software (v25, IBM, Armonk, NY) and R statistical software (v3.6.2, The R Foundation for Statistical Computing, Vienna, Austria), respectively, using a critical alpha value of 0.05.

## Results

Participants included 13 non-impaired controls (7 female/6 male, 29±11 years, 65.3±9.7 kg, 1.68±0.07 cm) and 6 BKPUs (5 female/1 male, 2 dysvascular/4 traumatic etiology, 48±8 years, 70.2±11.3 kg, 1.65±0.07 cm) using their customary prostheses. The BKPUs (additional characteristics in Table 1) all walked with a non-articulated dynamic prosthetic foot and comfortable sockets during testing, were experienced and regular (7 days/week) prosthesis users, and demonstrated high levels of functional balance. The perturbation magnitude was reduced to 10% bodyweight for one BKPU participant who was not comfortable with the 12% magnitude, and these data were included in the analysis as the perturbations delivered an observable destabilizing effect that was confirmed by the participant. All participants completed the full set of testing blocks. While only 6 BKPUs are included in the analysis, 8 were enrolled but 2 were unable to complete the study protocol, and this sample was further limited due to the narrow recruitment criteria seeking relatively capable individuals.

**Table 1:** BKPU participant characteristics (Median (interquartile range)) [26].

*Time since amputation (years)*	14.0 (11.3–17.5)
*Prosthesis use experience (years)*	13.0 (11.0–17.3)
*Prosthesis use frequency (hours/day)*	15.8 (12.9–16.0)
*Berg Balance Scale (ordinal scale 0–56)*	55 (54–56)
*Socket Comfort Score (ordinal scale 0–10)*	9 (8–10)

**Fig 2:**
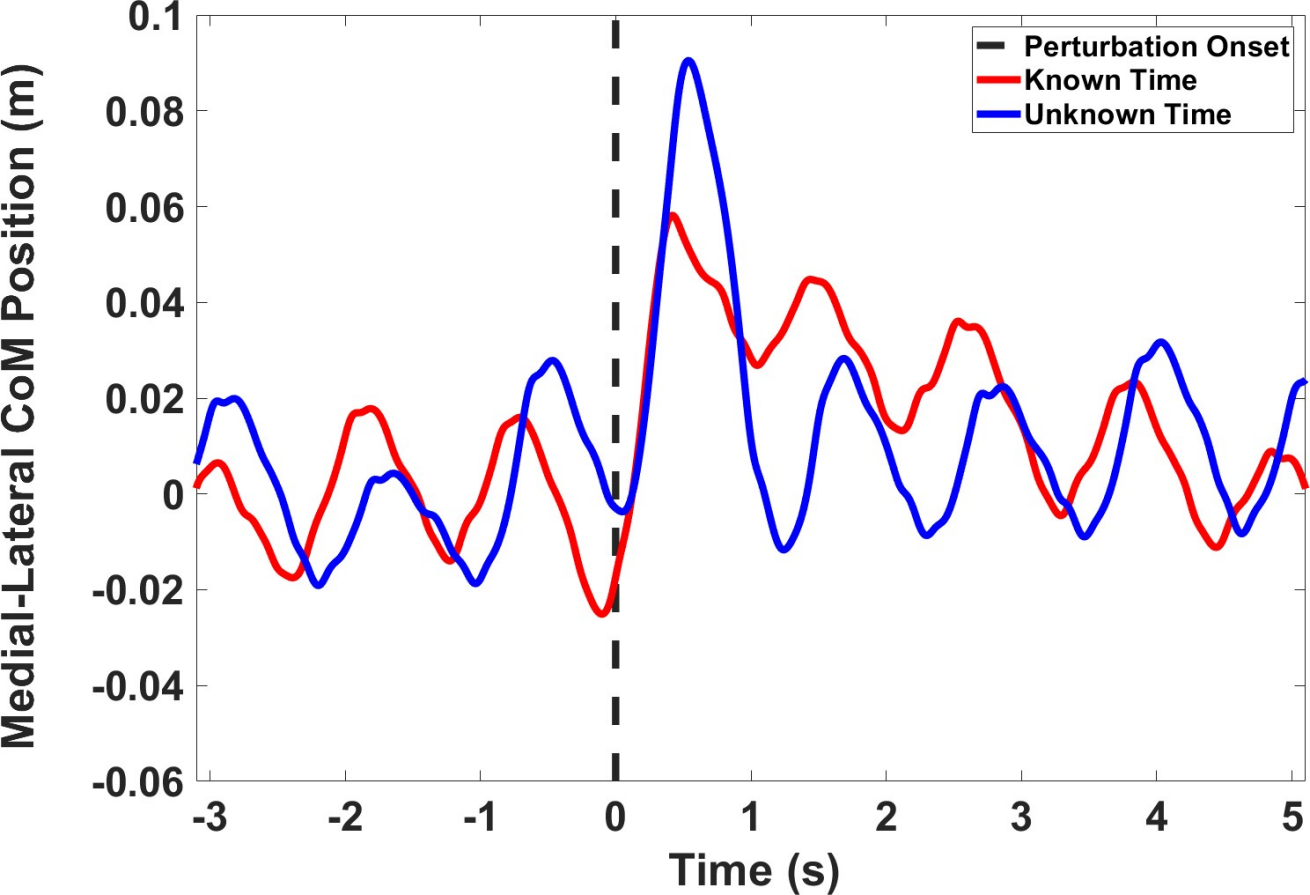
**Representative plots of temporal medial-lateral CoM position for a single BKPU subject perturbed toward the impaired limb (positive CoM position values) when the perturbation direction was known and the perturbation timing was either unknown (blue) or known (red).** The vertical dotted black line denotes the onset time of the perturbation (time 0) and the y-axis zero denotes the treadmill belt center.

The average walking speeds (±SD) for controls and BKPUs were 1.3±0.1 m/s and 0.8±0.3 m/s, respectively. The perturbations noticeably destabilized all participants, but none experienced a failed recovery (i.e., falls, or required stoppage or change in treadmill belt speed). Recovery was achieved through either side or cross-over stepping strategies (see Supplementary Data videos in Major et al 2018 [26]). Plots of representative temporal CoM position for a single BKPU subject are presented in Fig 2.

**Fig 3:**
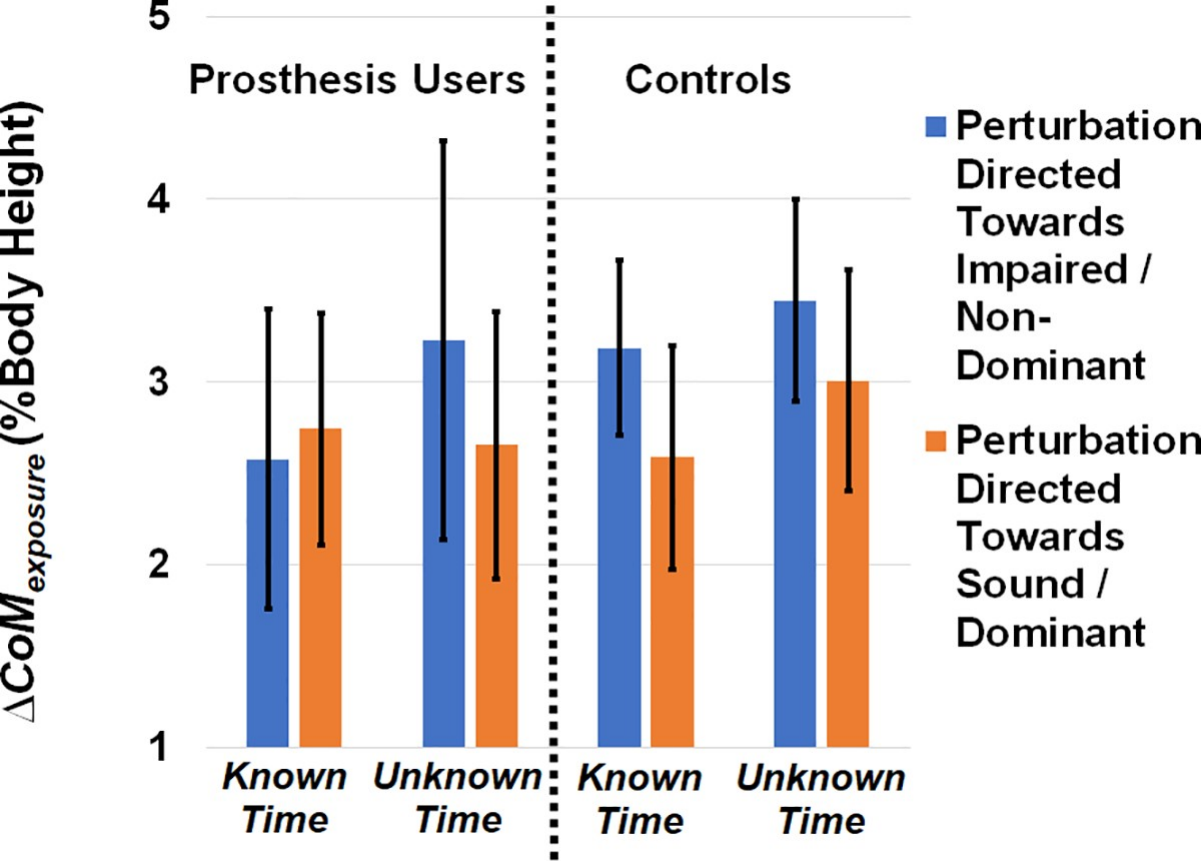
Average CoM displacement during the 400 msec exposure period (*ΔCoM*_*exposure*_*)* separated by group, perturbation direction, and perturbation timing knowledge. The main effect of perturbation direction was significant (p = 0.033) with displacement greater with perturbations towards the impaired/non-dominant side. Error bars denote 95% confidence intervals.

**Fig 4:**
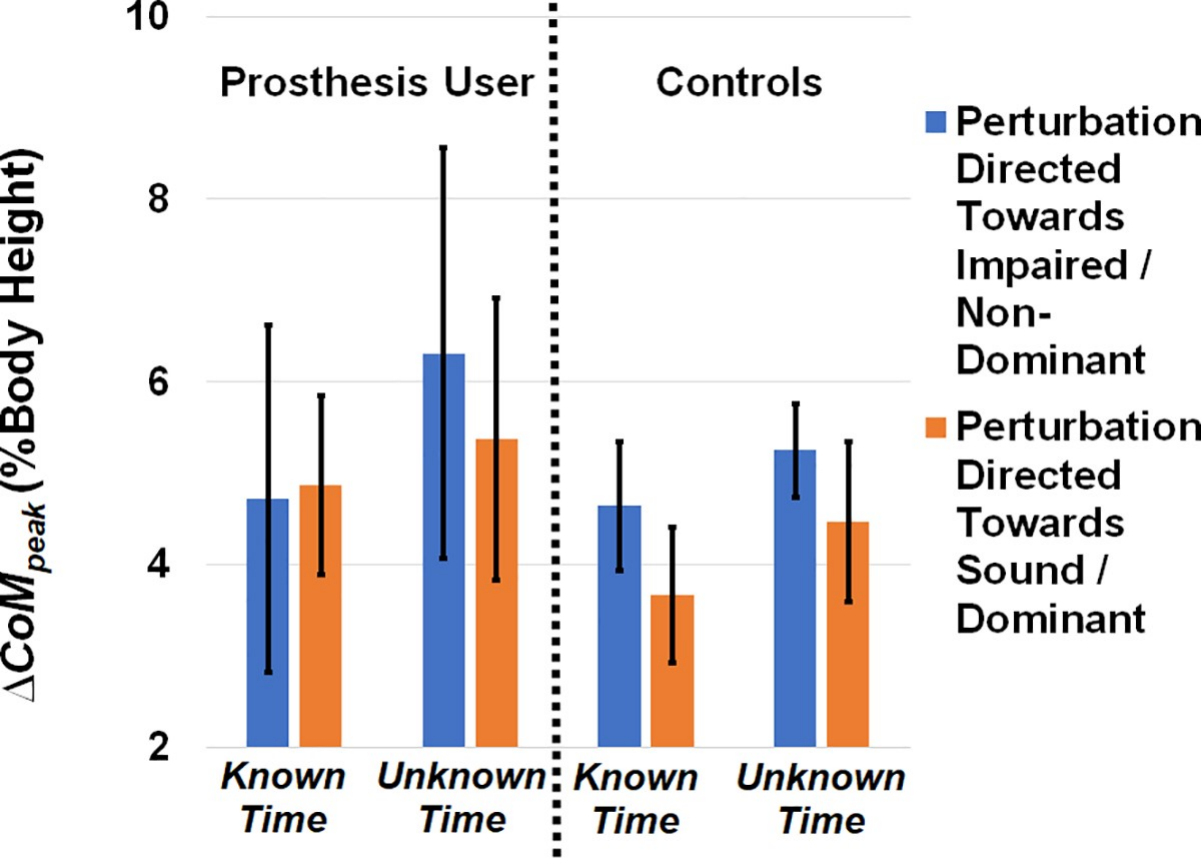
Average maximum CoM displacement (*ΔCoM*_*peak*_*)* separated by group, perturbation direction, and perturbation timing knowledge. The main effect of timing knowledge was significant (p = 0.010), with less displacement when perturbation timing was known. Error bars denote 95% confidence intervals.

**Fig 5:**
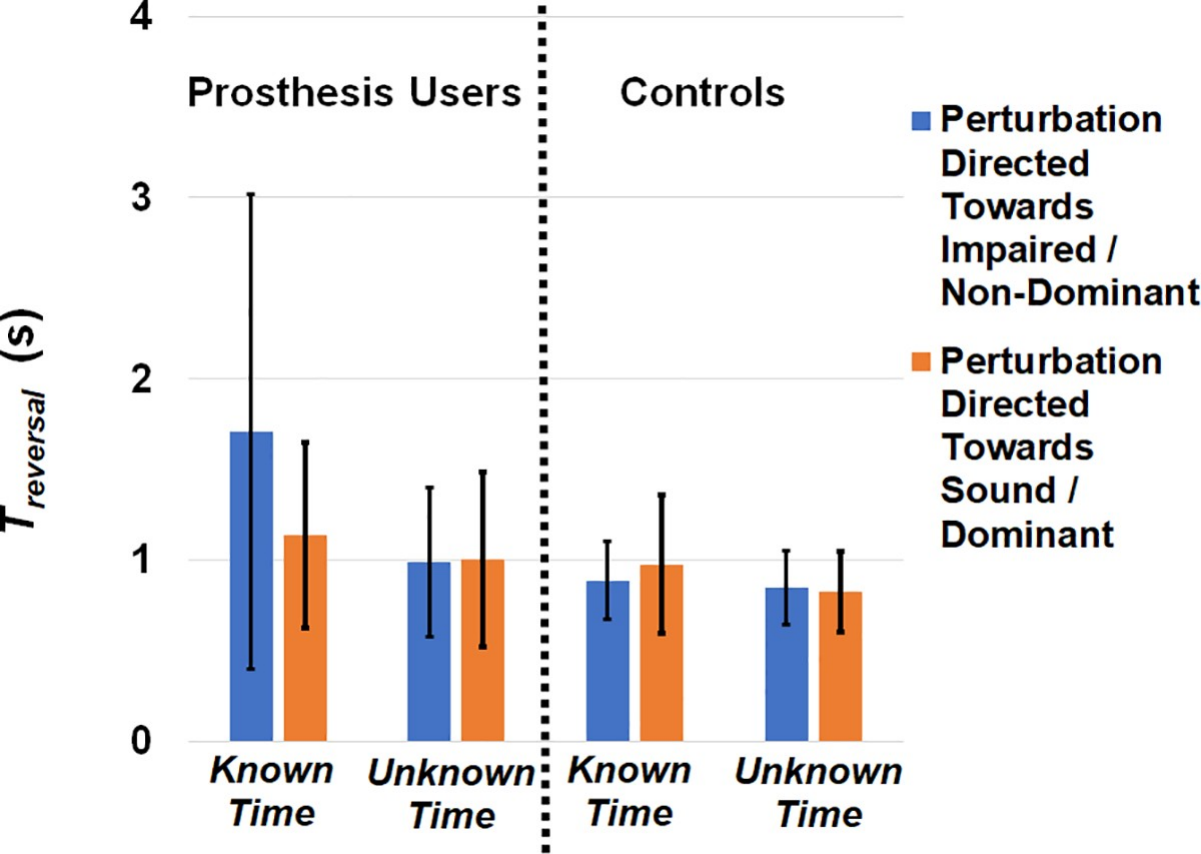
Average time to reversal (*T*_*reversal*_*)* separated by group, perturbation direction, and perturbation timing knowledge. The main effect of timing knowledge was significant (p = 0.043), with a greater *T*_*reversal*_ when perturbation timing was known. Error bars denote 95% confidence intervals.

**Fig 6:**
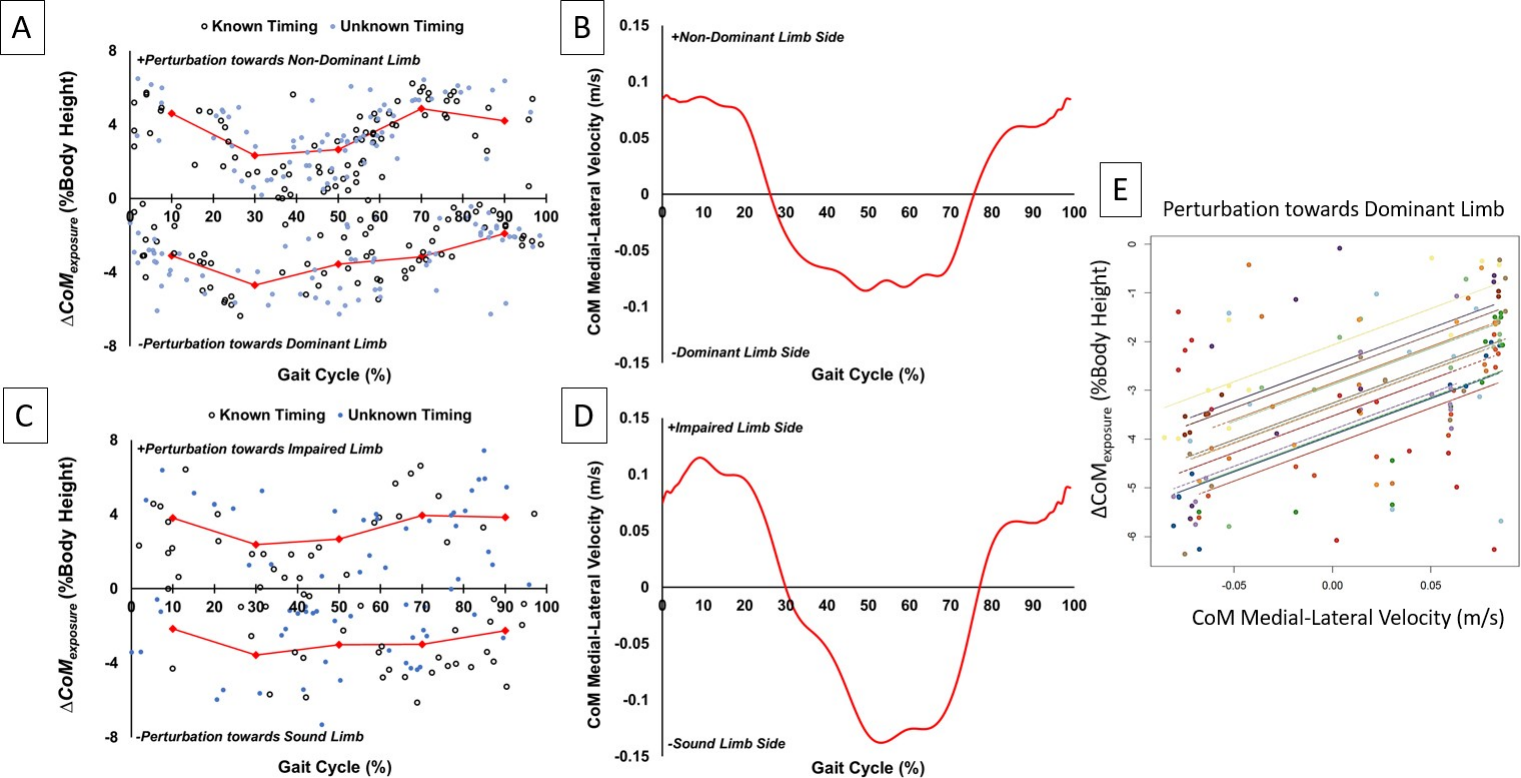
Plots of *ΔCoM*_*exposure*_ and average CoM medial-lateral velocity against gait cycle for control (A and B, respectively) and BKPU (C and D, respectively) participants. The connected red diamonds in plots A and C represent average displacement values for each 20% of the gait cycle to demonstrate how displacement varies over the gait cycle and has a similar shape to CoM velocity (B and D). Start of the gait cycle (0%) represents non-dominant or impaired limb initial contact. A representative scatterplot generated from the repeated-measures correlation analysis (E) demonstrates the positive linear relationship between CoM velocity and displacement for each able-bodied participant (denoted by different colors).

Average *ΔCoM*_*exposure*_, *ΔCoM*_*peak*_, *and T*_*reversal*_ are displayed in Figs 3, 4 and 5, respectively. Plots of *ΔCoM*_*exposure*_ versus gait cycle for controls and BKPUs are presented in Fig 6, with the average medial-lateral CoM velocity of each group displayed alongside for visual comparison. For all data reporting, the impaired/sound limb refer to the BKPUs, and the non-dominant/dominant limb refer to the controls.

For *ΔCoM*_*exposure*_, (Fig 3) the main effect of perturbation direction was significant (F(1,17) = 5.380, p = 0.033, partial η^2^ = 0.240), with displacement greater with perturbations towards the impaired/non-dominant side, but the main effect of group, main effect of timing, and interaction effects were not significant (F(1,17)≤1.541, p≥0.231, partial η^2^≤0.083).

For *ΔCoM*_*peak*_, (Fig 4) the main effect of timing knowledge was significant (F(1,17) = 8.421, p = 0.010, partial η^2^ = 0.331), with less displacement when perturbation timing was known, but the main effect of group, main effect of direction, and interaction effects were not significant (F(1,17)≤2.933, p≥0.105, partial η^2^≤0.147).

For *T*_*reversal*_, (Fig 5) the main effect of timing knowledge was significant (F(1,17) = 4.778, p = 0.043, partial η^2^ = 0.219), with more time when perturbation timing was known, but the main effect of group, main effect of direction, and interaction effects were not significant (F(1,17)≤1.912, p≥0.185, partial η^2^≤0.150).

To note, secondary three-way ANOVAs were performed on these data with a randomly selected subset of six participants from the able-bodied sample to create a balanced design. The results from this secondary analysis confirmed the significant main effects of perturbation direction and timing knowledge on *ΔCoM*_*exposure*_ and *ΔCoM*_*peak*_, respectively, without significant interaction effects, but found but no significant main or interaction effects on time to reversal. While key statistical values have been presented, findings should be interpreted given these collective results.

The repeated-measures correlation coefficients (r) evaluating the relationship between *ΔCoM*_*exposure*_ and unperturbed CoM medial-lateral velocity (Fig 6) at corresponding gait cycle times are presented in Table 2. All relationships were significant (p≤0.001), positive and strong according to Cohen’s effect size convention [35], with the exception of the condition of BKPUs perturbed towards the sound limb which was of low strength and marginally significant (p = 0.054).

**Table 2:** Repeated-measures correlation analysis results evaluating relationships between *ΔCoM*_*exposure*_ and CoM medial-lateral velocity.

Condition	r	p value
Non-impaired control, perturbation towards non-dominant limb	0.67	<0.001
Non-impaired control, perturbation towards dominant limb	0.59	<0.001
BKPU, perturbation towards impaired limb	0.50	<0.001
BKPU, perturbation towards sound limb	0.25	0.054

## Discussion

This study explored the effects of direction and *a priori* knowledge of onset timing of a lateral perturbation on the CoM response dynamics of BKPUs and non-impaired controls. We hypothesized that BKPUs would exhibit a greater lateral CoM displacement when perturbations directed toward their impaired limb occurred, irrespective of timing knowledge, when compared to the effects of perturbations directed toward their sound limb. Further, we hypothesized that due to the additional anticipatory control strategies observed in BKPUs when perturbations of known timing were possibly directed toward their impaired limb, there would be smaller CoM displacements resulting from known timing perturbations than unknown time perturbations. These hypotheses were generally supported when considering both groups and depended on the CoM displacement metric, but no group or interaction effects were significant to suggest that responses were not specific to BKPUs.

We found that *ΔCoM*_*exposure*_, the acute displacement of the CoM during the period of perturbation exposure, was greater when BKPUs and control participants were perturbed towards the impaired/non-dominant limb respectively (Fig 3). In the case of BKPUs this increase in excursion when perturbed toward the impaired limb could be expected due to the compounded effects of reduced sensory feedback and active joint control on this limb [1, 6–10, 36], which would compromise the ability to quickly generate stance-limb ankle torque center-of-pressure corrections in response to a sudden disturbance. There was no observable effect of *a priori* perturbation timing knowledge, indicating that a larger *ΔCoM*_*exposure*_ when directed towards the impaired or non-dominant limb occurred irrespective of whether or not the onset of the perturbation was predictable. However, previous results have indicated that BKPUs enact anticipatory locomotor adjustments when the onset timing is known by increasing the MoS on the impaired limb side prior to the perturbation [26]. In light of these results, this strategy may protect BKPUs against the observed larger CoM displacement exceeding the lateral base-of-support which would theoretically require a risky corrective step [26, 37].

An unexpected result was that there was no difference in *ΔCoM*_*exposure*_ between groups, suggesting that controls also experienced greater CoM displacement toward their non-dominant side and this bias seemed more consistent than BKPUs (Fig 3). This result seems to align with some literature on limb laterality and the natural functional differences between the dominant and non-dominant limbs during gait, specifically that the non-dominant limb is more responsible for stability control whereas the dominant limb contributes more to propulsion [38]. One interpretation may be that non-impaired individuals are willing to accept greater CoM excursion towards the non-dominant limb given its functional role as a gait stabilizer. Although not mutually exclusive, this body of literature also suggests that the non-dominant side is the weaker side [38] and so an alternative interpretation is that reduced muscle strength yields greater CoM displacement when perturbed towards that side. As the functional implications of limb dominance are still in debate, the contribution of limb laterality to asymmetrical response to lateral perturbations should be further explored.

Our results suggest that maximum displacement of the CoM following perturbation onset is reduced for BKPUs and non-impaired controls when onset time is provided *a priori* (Figs 2 and 4). Importantly, as timing knowledge did not significantly affect *ΔCoM*_*exposure*_, the results suggest that *a priori* knowledge influence is specific to maximum CoM displacement. As there was no observable effect of perturbation direction, this suggests that BKPUs and controls are equally capable of limiting the *ΔCoM*_*peak*_ on both sides when the perturbation onset time can be predicted. Specific to BKPUs, this result aligns with our previous study suggesting that anticipatory locomotor adjustments for BKPUs is time-dependent, in which knowledge of the perturbation time may enable an individual to make anticipatory adjustments so that modifiable factors including; body mechanics and phase in the gait cycle, can be modulated to mitigate the effects of the perturbation at the moment it occurs [26]. Due a lack of observable difference between groups, this finding also includes the non-impaired participants in which the ability to predict perturbation onset also yielded a reduced CoM excursion. Since controls did not exhibit detectable anticipator locomotor adjustments when timing is provided *a priori* [26], it may be that other neuromuscular adaptations such as increased joint impedance [39, 40] were utilized to prepare for the perturbation that were not captured in this study and this possibility should be explored in future investigations. Furthermore, timing knowledge also appeared to increase *T*_*reversal*_, suggesting that the ability to predict perturbation onset facilitates other adjustments that permit a longer response time until the CoM is redirected back towards center. One interpretation is that a longer delay, or ‘riding out’ the disturbance as opposed to a more rapid response, may be acceptable when the risk is reduced due to increased contextual knowledge [41]. Although jointly these finding suggest that the ability to predict an impending perturbation can influence CoM spatiotemporal response dynamics, it is important to note that both scenarios are recoverable given the perturbation magnitude delivered in this study. Research should also explore the effects of timing knowledge on reactive control with larger disturbances.

Addressing the secondary aim of characterizing the effects of gait cycle timing on *ΔCoM*_*exposure*_ (Fig 6) revealed two observations. The magnitude of *ΔCoM*_*exposure*_ with perturbations toward the impaired/non-dominant limb was the smallest during single-limb support on the impaired/non-dominant limb and increased during single-limb support on the sound/dominant limb. This may partially explain why a previous study identified that when perturbed towards the impaired/non-dominant limb [26], controls/BKPUs were less likely to be in sound/dominant single-limb support possibly as a means to avoid experiencing larger CoM disturbances. Secondly, the strength of this relationship was quantified through a correlation analysis and generally suggested a positive association between *ΔCoM*_*exposure*_ and corresponding instantaneous CoM medial-lateral velocity for both BKPUs and controls (Table 2). This finding suggests that even instantaneous CoM medial-lateral velocity during separate unperturbed walking trials demonstrates some relationship with the CoM response dynamics during the perturbation trials. The association can be defined by: larger CoM medial-lateral velocity was related to greater acute CoM response excursion when both the perturbation and velocity were in the same direction. Given this relationship, it is possible that when the timing of perturbation was known, individuals may have employed a multi-step preparatory strategy so that the point in the gait cycle when the perturbation occurred aligned with instances when the CoM medial-lateral velocity was small or directed in the opposite direction of the perturbation. This association supports the theoretical relationship between CoM velocity (and hence body momentum) and frontal-plane balance control [36, 42], and also highlights regions of the gait cycle to target when the desire is to implement challenging disturbances for stability training interventions [43].

There are certain study limitations to consider when interpreting these results. First, the perturbation duration (exposure) was 400 msec. While this perturbation period was used to generate an acute disturbance, this feature also made it difficult to isolate the effects of the perturbation to a specific instant in the gait cycle. Second, the study design involved random delivery of perturbations during the gait cycle and so the timing of the disturbance relative to cycle was not controlled. Consequently, the secondary aim of evaluating the relationship between gait-cycle dependent COM velocity and perturbation response could only be achieved through the available data and not a systematic sweep of cycle time. Fourth, the relatively small BKPU sample size limited the statistical power of this study, and therefore may have reduced ability to detect group differences. Moreover, the response behavior findings in this study resulted from analyses across groups and were not specific to BKPUs to directly support or reject the stated hypotheses. However, these results revealing significant main effects of perturbation direction and knowledge of timing but non-significant group effects suggest directions for future investigation on this topic but with larger BKPU sample sizes for greater statistical power. Furthermore, the tested sample of community-dwelling BKPUs demonstrated high levels of baseline balance function, and the results are therefore limited by the representativeness of cohort. However, fall risk is relevant for prosthesis users at all stages in the rehabilitation journey [13], including community-dwelling individuals, and so these findings remain relevant to the body of work on this topic. Finally, the result should be interpreted knowing that there is potential for a learning effect given the repeated exposure of perturbations.

The results of this work have particular clinical implications. The greater COM displacement on the impaired and non-dominant limb side during perturbation exposure might suggest greater potential to fall towards that side. For BKPUs particularly, a greater excursion of the COM when ankle strategies are lacking would require some alternative motor strategy to maintain balance and possible reliance on hip musculature [44]. Furthermore, the finding that peak COM displacement was smaller when timing of the perturbation was known and there was no difference between limbs (perturbation direction) suggests that contextual awareness of the surrounding environment may be critical to preventing falls. Although seemingly evident, maintaining focused attention on a constantly changing environment and recognizing potential sources of balance disturbance are not trivial, especially as BKPUs monitor the ground to facilitate safe foot placement of their impaired limb [45]. Finally, our combined work on this topic of anticipatory and reactive strategies to respond to a medial-lateral perturbation during walking reemphasizes that to prepare individuals for walking disturbances and hence mitigate fall risk, it may be important to practice responses to both known and unknown perturbations given the different recovery strategies for each. These results may suggest future study designs to assess the effectiveness of varied medial-lateral perturbations on reducing fall occurrence.

## Conclusions

This study observed the effects of direction and *a priori* timing knowledge of a medial-lateral perturbation on response behavior of BKPUs and non-impaired controls. The results suggest that the displacement of the COM during the short exposure period of the perturbation was greater on the impaired or non-dominant limb for BKPUs and controls, respectively. However, peak COM displacement was reduced for both cohorts when the timing of the perturbation is known, which also increased the timing delay between perturbation onset and redirection of the COM back towards center. Importantly, medial-lateral COM displacement during perturbation exposure appears to be coupled with instantaneous medial-lateral COM velocity during the gait cycle. Knowing how these factors (*a priori* knowledge and gait cycle timing) affect response behavior may facilitate the design of perturbation paradigms to train for improved locomotor stability.
